# Drilling Process in γ-TiAl Intermetallic Alloys

**DOI:** 10.3390/ma11122379

**Published:** 2018-11-26

**Authors:** Aitor Beranoagirre, Gorka Urbikain, Amaia Calleja, Luis Norberto López de Lacalle

**Affiliations:** 1 Department of Mechanical Engineering, University of the Basque Country (UPV/EHU), Plaza Europa 1, 20018 San Sebastián, Spain; 2 Department of Mechanical Engineering, University of the Basque Country (UPV/EHU), Nieves Cano 12, 01006 Vitoria, Spain; amaia.calleja@ehu.eus; 3 CFAA, University of the Basque Country (UPV/EHU), Parque Tecnológico de Zamudio 202, 48170 Bilbao, Spain; norberto.lzlacalle@ehu.es

**Keywords:** Gamma-TiAl, superalloys, slight materials, drilling, titanium aluminides

## Abstract

Gamma titanium aluminides (γ-TiAl) present an excellent behavior under high temperature conditions, being a feasible alternative to nickel-based superalloy components in the aeroengine sector. However, considered as a difficult to cut material, process cutting parameters require special study to guarantee component quality. In this work, a developed drilling mechanistic model is a useful tool in order to predict drilling force (*F_z_*) and torque (*T_c_*) for optimal drilling conditions. The model is a helping tool to select operational parameters for the material to cut by providing the programmer predicted drilling forces (*F_z_*) and torque (*T_c_*) values. This will allow the avoidance of operational parameters that will cause excessively high force and torque values that could damage quality. The model is validated for three types of Gamma-TiAl alloys. Integral hard metal end-drilling tools and different cutting parameters (feeds and cutting speeds) are tested for three different sized holes for each alloy.

## 1. Introduction

Aeronautics is an emerging sector; aircraft manufacturing predictions estimate doubling of the current fleet by the year 2033, in order to satisfy passenger’s increment demand that grows at a rate of 4.2% per year [1]. Components manufacturing for the aeronautic sector is a high value added process, and, concretely, motor components require special attention because they represent one of the most expensive (20%) components [2]. The aviation industry will try to satisfy manufacturing demand according to regulation requirements regarding efficiency, noise, fuel consumption and contamination. Therefore, new materials and manufacturing processes are under development by aeronautic sector manufacturers. In this sense, γ-TiAl alloys are a feasible replacement for nickel-based alloys frequently used for compressor blades and stator in gas turbine aeroengines [3,4,5]. Moreover, titanium alloys are also interesting for bio-medical engineering, automobile sector and chemical industries [6].

Gamma titanium aluminide intermetallics present excellent strength-weight ratio, and corrosion resistance at high temperature [7,8]. However, it is considered a difficult to cut material [9,10] due to its poor tensile and low room temperature ductility (<2%). Besides, high heat values are generated during γ-TiAl machining due to low thermal conductivity o the material. Consequently, tool and workpiece wear is accelerated [11], tool life is reduced [12] and workpiece integrity is affected [13]. Moreover, γ-TiAl reacts chemically with many materials causing material adhesion.

Several studies have considered γ-TiAl a difficult to cut material regarding turning [14,15], grinding [16], high speed milling [17,18,19], drilling and micro-drilling [20].

In this sense, high machining conditions can cause irreversible consequences such as surface cracking, hardened layers and tensile residual stresses [21]. Optimal machining strategies and parameters are studied for electrochemical machining [22], turning [23,24], and milling [25] related to cutting temperature technique evaluation, and, appropriate machining conditions in order to reduce tool wear and increase tool life. Milling studies have also investigated the effects of operating parameters and conditions on tool life and surface integrity when milling γ-TiAl alloys [26,27,28].

In relation to lubrication techniques, some studies [29] focus on cryogenic lubrication (liquid nitrogen) obtaining cutting feed value increments while tool life is maintained.

Machining processes modelling is also a hot topic for difficult to cut materials. In this sense, two-dimensional models [30] were developed for cutting parameters such as cutting speed and feed influence determination. Numerical and experimental analyses of residual stresses generated in the machining of Ti6A14V titanium alloy are developed [31]. Empirical models are also used for thrust and torque values prediction in composites. Other techniques such as response surface methodology (RSM) and finite element analysis (FEA) are implemented for milling [32] and drilling [33] process parameter analysis. In addition, finite element models are also developed for performance characteristics such as hole quality prediction [34]. Other techniques are focused on the side of monitoring. For instance, power consumption during drilling is a key magnitude in predictive/preventive techniques [35,36].

Considering γ-TiAl promising application possibilities, drilling machinability studies are a focus of interest due to presented machinability problems, drilling being one of the most frequent processes for component assembly. In this work, the developed drilling mechanistic model is a useful tool in order to predict drilling force (*F_z_*) and torque (*T_c_*) for optimal drilling conditions determination. The model is validated for three types of Gamma-TiAl alloys. Integral hard metal end-drilling tools and different cutting parameters (feeds and cutting speeds) are tested for three different sized holes for each alloy.

## 2. Experimental Procedure

### 2.1. Material

Four different titanium alloys are tested in performed drilling tests: Ti-6Al-4V and three different γ-TiAl alloys (TNB, extruded MoCuSi and ingot MoCuSi). Table 1 shows the tested alloys’ mechanical properties.

γ-TiAl alloys present higher aluminum percentage in comparison to other titanium alloys such as Ti-6Al-4V, 43–48% in γ-TiAl and 6% in Ti-6Al-4V, improving thermal conductivity in γ-TiAl. On the other hand, ductile transition temperature occurring between 600 and 800 °C, depending on the microstructure and grain size alloys, is increased for alloys with higher titanium percentage [12]. The intermetallic TiAl provides low density [37] as well as high mechanical strength under high temperatures and corrosive environments. The intermetallic γ-TiAl superalloys offer excellent mechanical properties [38], with low density (4 gr/cm^3^), high resistance at high temperatures, low electrical and thermal conductivity, oxidation resistance, ultimate strength of 1000 MPa and Young’ s modulus of 160 GPa.

MoCuSi alloy [Ti-(43–46) Al-(1–2) Mo-(0.2) Si-Cu] is used at low temperatures with high resistance below 650 °C, and, TNB alloy [Ti-(44–45) Al-(5–10) Nb-(0.2–0.4) C] resists very high temperatures maintaining high resistance and oxidation values. Regarding MoCuSi alloys, the material is presented in both, extruded and ingot structure. Extruded alloys present an oriented structure oriented in the extrusion direction whereas melted alloys present a structure without any preferable orientation, typical of no extruded or laminated materials. The ingot structure is directly obtained from the VAR (Vacuum Arc Remelting) process. For extruded structures, material is extruded at 1200 °C and smaller sized grains are obtained with superior creep strain, yield strength and KIC.

### 2.2. Equipment

**Figure 1:**
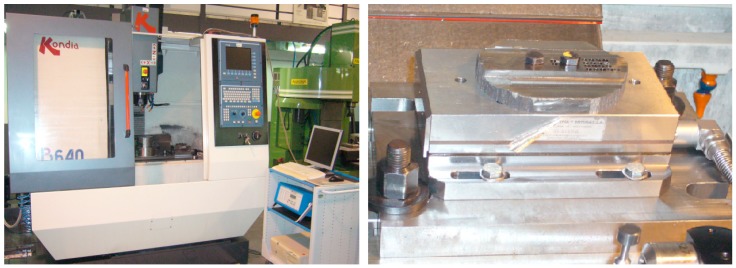
Kondia^®^ model B640 (**left**) and Kistler^®^ 9257B dynamometer (**right**).

Machining tests were performed in a vertical CNC milling machine, Kondia^®^ model B640 (Kondia, Elgoibar, Spain), with maximum rotational speed of 10,000 rpm and 25 kW. During the tests, process-cutting conditions are measured and recorded. For the axial/thrust (*F_z_*) and radial (*F_x_*, *F_y_*) cutting forces, and, Z axis (*T_c_*) torque measurements, a dynamometric Kistler^®^ equipment 9257B (Kistler, Winterthur, Switzerland) was used (Figure 1).

### 2.3. Machining Conditions

**Figure 2:**
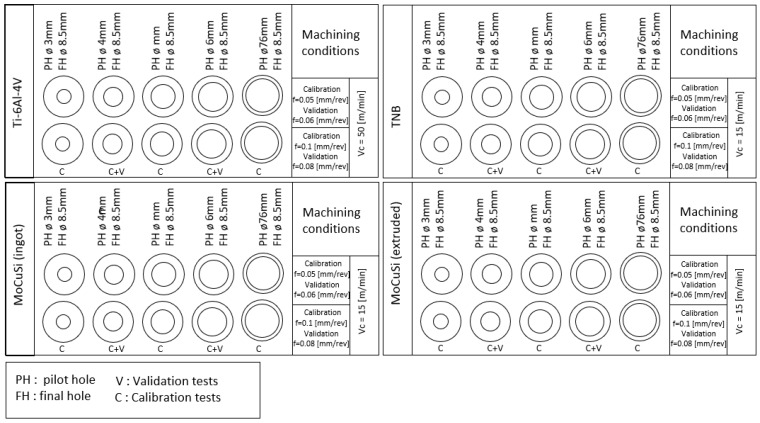
Experimental holes set–up.

Drilling tests were carried out for the described four titanium alloys (Table 1). As can be seen in Table 2, for each material, tested feed values are 0.05 and 0.1 mm/rev for calibration tests and 0.06 and 0.08 mm/rev for validation tests, and, *D* = 3, 4, 5, 6, 7 mm (pilot holes) and 8.5 mm holes (final holes) were drilled in each case (Figure 2). The drilled depth is 20 mm. *D* = 3, 4, 5, 6, 7 mm (pilot holes) and 8.5 mm holes (final holes) are used for calibration, and, *D* = 4–6 mm (pilot holes) and 8.5 mm holes (final holes) are used for the validation tests.

**Figure 3:**
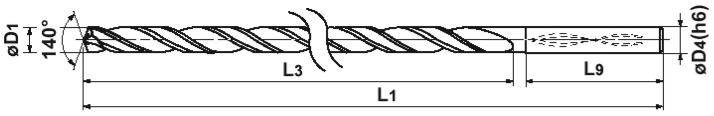
Cutting tool geometry.

The selected tool is a solid carbide drill (Mitsubishi^®^, Tokyo, Japan, MPS0850S-DIN-C, Figure 3). During the machining operations, cutting forces, torque and power consumption were recorded.

One of the critical aspects when drilling γ-TiAl is chip evacuation and heat dissipation. Internal lubrication is directly applied on the cutting edge, due to the poor thermal conductivity of these alloys. Specially designed for low machinability materials, Rhenus^®^ FU W (Table 3, Rhenus, Mönchengladbach, Germany) coolant was used. Internal coolant pressure is set to 8.5 bar.

## 3. Cutting Forces Prediction Mechanistic Model

Predictive cutting forces mechanistic models help programmers with cutting parameter selection. Especially when drilling low machinability titanium alloys, it is interesting to simulate different machining conditions in order to decide whether the estimated cutting forces values lead to an optimum machining process.

This section explains the modelling of cutting forces and torque in drilling. Since the cutting speed varies along the drill’s lips, the way the cutting force coefficients are introduced inside the model is a crucial issue. There have been a number of attempts based on orthogonal-oblique transformation [39] and mechanistic models [40].

**Figure 4:**
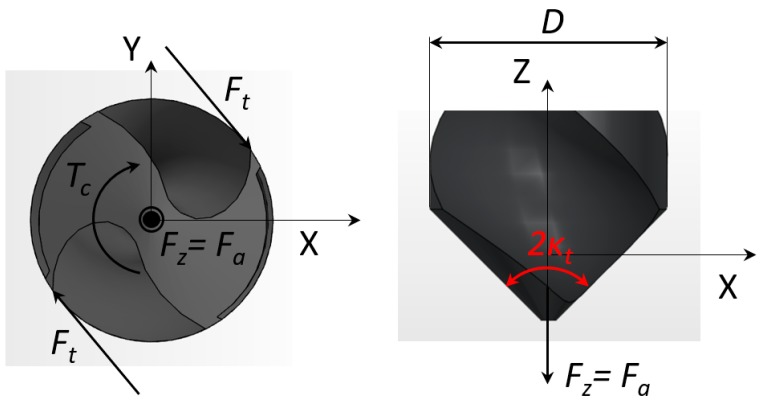
Cutting or tangential force (*F_t_*), thrust force (*F_z_*) and cutting torque (*T_c_*) in the drilling process; Drill bottom view (**left**) and front view (**right**).

The cutting edge is divided into discrete elements in the drill axis (*j* = 1 to *n* elements) and the global cutting force and torque are obtained by summing the elemental contributions along each lip (*i* = 1 to *Z*). As it can be seen in Figure 4, while *F_x_* and *F_y_* are coupled due to tool rotation leading to the radial *Fr* and tangential Ft cutting force components, *F_z_* is directly obtained from the axial component *Fa*. Here, we will focus on the thrust force (Equation (1)) in *Z* direction and torque (Equation (2)), which are critical parameters when characterizing tool life. These magnitudes are calculated as:

(1) $$F_{z}\left(t\right)=\sum_{i=1}^{Z}\sum_{j=1}^{n}K_{c,z}\left(h,r\right)\cdot \Delta z\cdot h$$

(2) $$T_{c}\left(t\right)=\sum_{i=1}^{Z}\sum_{j=1}^{n}K_{t,z}\left(h,r\right)\cdot \Delta z\cdot h\cdot r$$

where *K_z_* and *K_T_* are the corresponding thrust force and torque coefficients, Δ*z* is the axial width of a differential element, h the chip thickness (here, *h = 0.5f·sin70°*) and *r* the tool radius. As usual, the thrust cutting force and torque coefficients need to be calibrated. Here, 4 different titanium alloys were tested: (1) Ti-6Al-4V, taken as reference; (2) TNB and MoCuSi types, (3) extruded and (4) ingot types. Under this approach, the cutting force coefficients were assumed a function of the feedrate (or h) and radial distance of the cutting point to drill axis as quadratic functions. First, *a*_0_-*a*_5_ and *b*_0_-*b*_5_ coefficients in Equations (3) and (4) are identified from a linear regression for the four types of materials from a set of experiments.

(3) $$K_{z}\left(h,r\right)=a_{0}+a_{1}\cdot h+a_{2}\cdot r+a_{3}\cdot hr+a_{4}\cdot r^{2}+a_{5}\cdot h^{2}$$

(4) $$K_{T}\left(h,r\right)=b_{0}+b_{1}\cdot h+b_{2}\cdot r+b_{3}\cdot hr+b_{4}\cdot r^{2}+b_{5}\cdot h^{2}$$

## 4. Results

The mechanistic model is valid for *v_c_* = 50 m/min (Ti-6Al-4V), and *v_c_* = 15 m/min (TNB, and extruded/ingot types), the cutting speed at the tool diameter *D*. To identify the cutting coefficients, 10 tests were done. After a pilot hole, the hole was finished at *D* = 8.5 mm. Five different pilot hole diameters (*D*_0_ = 3, 4, 5, 6, 7 mm) and two feed rates (*f* = 0.05–0.1 mm) were programmed.

The corresponding functions for the cutting coefficients are summarized in Table 8 with the obtained coefficients of polynomial functions *K_z_* and *K_T_* in Equations (3) and (4).

### 4.1. Results for Ti-6Al-4V Alloy

**Figure 5:**
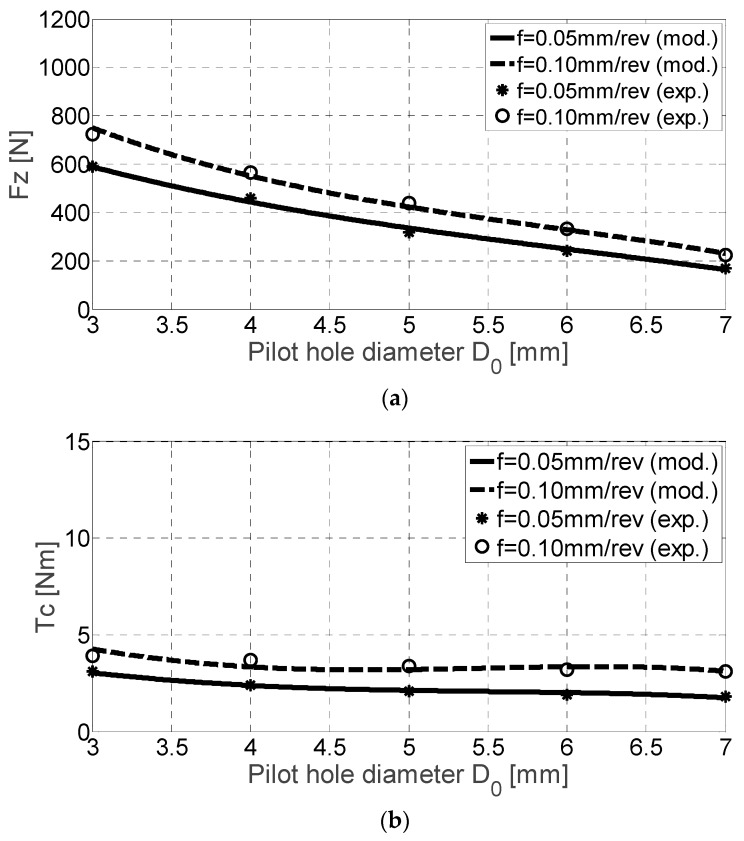
*F_z_* [N] (**a**) and *T_c_* [Nm] (**b**) for Ti-6Al-4V alloy drilling experimental and mechanistic model values.

As can be seen, both experimental and the predicted thrust force and torque values are higher for higher feed rate values (Figure 5). Table 4 shows the experimental data for both variables during the calibration of the cutting coefficients. While the curve *F_z_* reflects almost a linear tendency, the torque is approximated as a curve with an inflexion (third order). At the same time, the higher the dispersion, the lower is the difference between the pilot and final hole diameters.

### 4.2. Results for TNB Alloy

**Figure 6:**
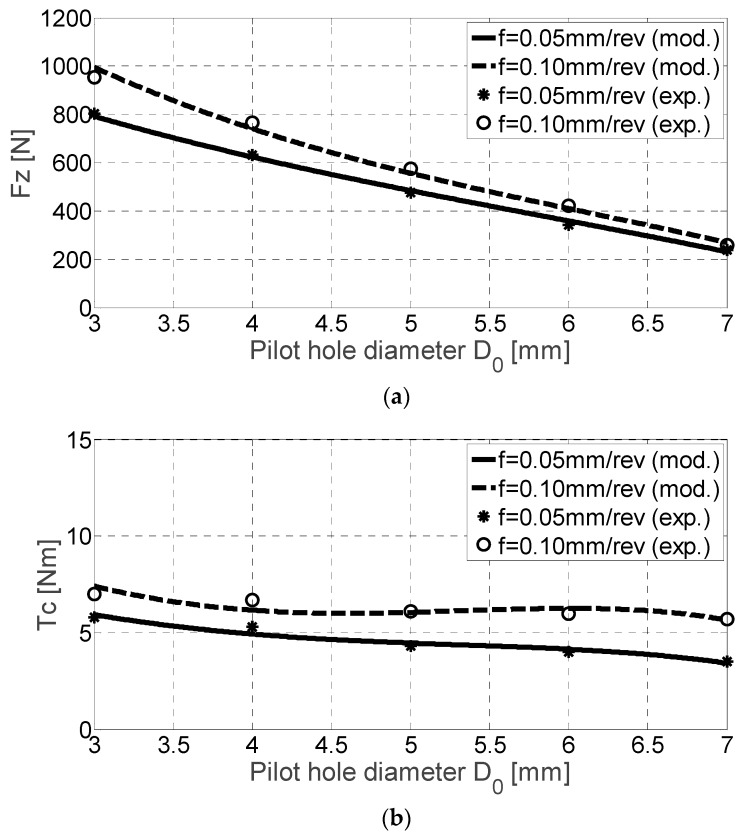
*F_z_* [N] (**a**) and *T_c_* [Nm] (**b**) for TNB alloy drilling experimental and mechanistic model values.

Thrust force values (Figure 6a) show higher values for higher feed rate values but are not doubled when doubling the feed rate. However, the cutting tool behaved correctly despite that the cutting force increased ×1.33 and cutting torque increased ×1.5–2, with respect to Ti4Al6V. When feed rates values are multiplied ×2, torque values are multiplied ×1.3 approximately. Table 5 shows again the experimental data necessary to obtain the polynomials of the cutting coefficients. A higher dispersion is seen for the cutting torque *T_c_* as the pilot hole diameter approaches the final hole diameter (Figure 6b).

### 4.3. Results for Ingot MoCuSi Alloy

**Figure 7:**
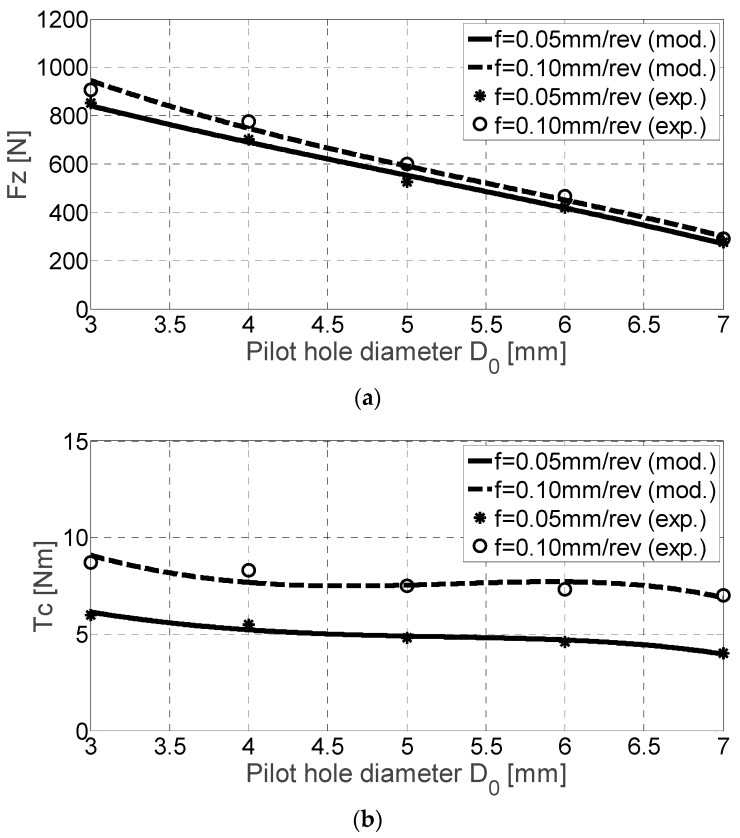
*F_z_* [N] (**a**) and *T_c_* [Nm] (**b**) for ingot MoCuSi alloy drilling experimental and mechanistic model values.

Ingot MoCuSi alloys are harder Ti-6Al-4V alloys and TNB alloys. Table 6 shows the main values. This statement was verified on sight of Figure 7. The axial force *F_z_* and cutting torque *T_c_* are higher regarding previous alloys. Focusing on cutting torque (Figure 7b), when the feed rate value is multiplied ×2, torque values are multiplied ×1.1 approximately.

### 4.4. Results for Extruded MoCuSi Alloy

**Figure 8:**
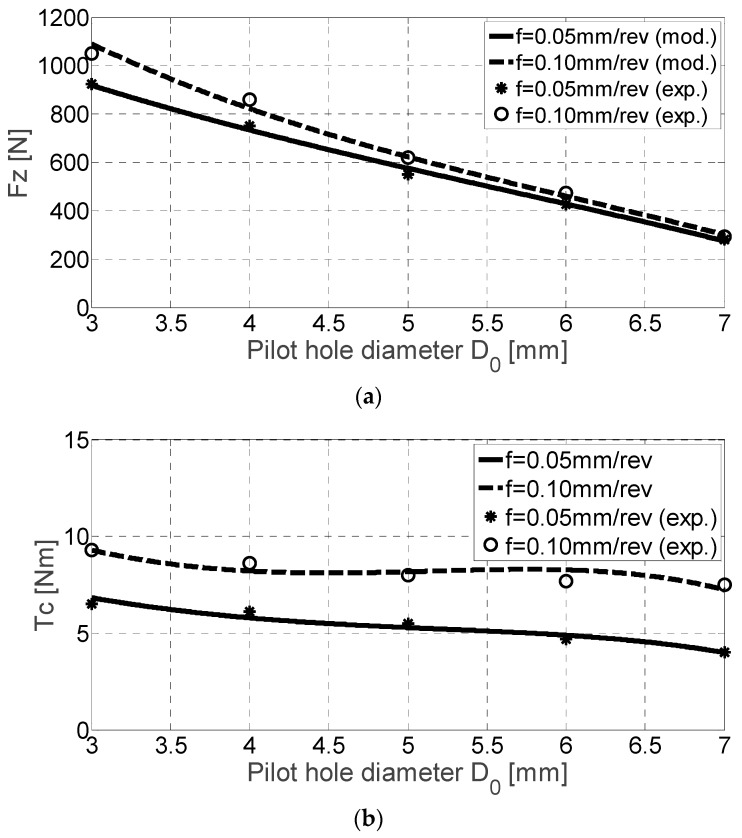
*F_z_* [N] (**a**) and *T_c_* [Nm] (**b**) for extruded MoCuSi alloy drilling experimental and mechanistic model values.

MoCuSi extruded alloys present the highest hardness values in comparison to the latter ones. While the results for the cutting torque *T_c_* are quite similar to the ingot MoCuSi alloy (both results are almost identical), the thrust force is more dramatic for the extruded MoCuSi alloy. Regarding cutting torque (Figure 8b), when the feed rate value is multiplied by two, torque values are multiplied ×1.2 approximately (see also results in Table 7).

Using the above experimental results, the coefficients in Equations (3) and (4) are obtained and then the corresponding polynomial functions are built (Table 8).

For model validation, different combinations of parameters were tested within the window parameters. New pilot hole drills and final drill of *D* = 8.5 mm were used for these tests. Eight different tests were programmed to put the model to work. Table 9 presents the conditions for these validation tests.

**Figure 9:**
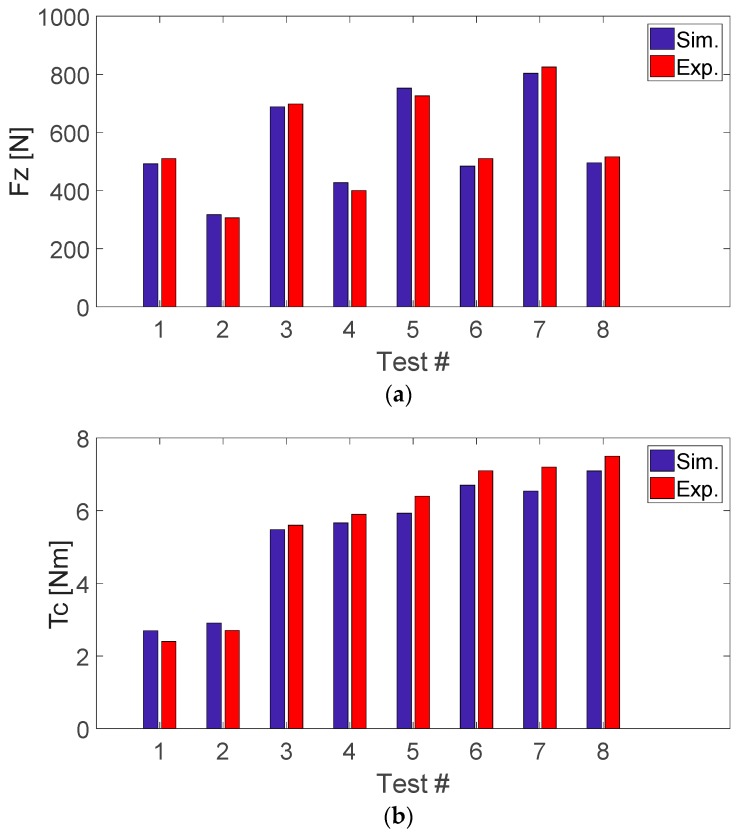
Model validation for *F_z_* (**a**) and *T_c_* (**b**) magnitudes.

Figure 9 shows the results from the simulated values using the model described and the experimentally measured values. The model presents a good correlation with the real thrust forces and cutting torques. For the cutting forces, the maximum relative error between the predicted and measured values was 8% while for the cutting torque the worst case was found to be 13%. The model behaves well in the targeted interval of feed rates, with higher deviations, the higher is the programmed feed. On the other hand, the mechanistic model seems less sensitive to the range of tested pilot hole diameters and final hole diameters. A higher dispersion is seen also in the torque values with respect to thrust forces that can be predicted with very good agreement.

## 5. Conclusions

This work presents the machinability results for four different titanium alloys. Currently there are few data about machining of γ-TiAl. The obtained results reflect that:

- These alloys present a much smaller machinability than conventional titanium alloys.
- The presented mechanistic stationary model is capable of simulating the thrust force and cutting torque in the drilling process. The influence of vibratory effects, lateral vibrations or runout was not considered. After model calibration, validation tests were done, showing a good agreement.
- Experimental drilling tests done to evaluate the machinability of these difficult-to-cut materials (the high cost of the tested materials (approx. 400 €/kg) limited the number of tests and a stronger experimental design) show that the obtained cutting coefficients present a quasi-linear dependence on the pilot hole *D*_0_ for the axial force *F_z_*, while a cubic is more suitable in the case of *T_c_*.
- Machining problems during drilling tests confirm that these are very brittle materials, so operational parameter determination is essential for chipping and cracking avoidance of components during machining processes [41,42].
- Experiments demonstrated large differences between the more conventional Ti6Al4V alloy and the three γ-TiAl intermetallic alloys. As the mechanical properties of extruded/ingot alloys are higher than those of the TNB alloy, this trend is inversely true for the machinability grade. Tests proved that MoCuSi alloys are the toughest materials.
- Despite the good results during the validation, a statistical approach would be desirable to test any differences in material series, machine tools, as well as to build more complete models. Wear studies including process damping effects can be subjects for future research works.

## Figures and Tables

**Table 1:** Mechanical properties comparison between TiAl alloys.

Property	TNB	Ti–6Al–4V (Annealed)	MoCuSi Extruded	MoCuSi Ingot
Density (g/cm^3^)	3.86	4.49	3.74	3.88
Specific modulus (GPa/(mg·m^−3^))	43	24	43	37
Tensile strength (MPa)	683	1087	607	689
Specific strength (MPa/(g·cm^−3^))	192	947	198	180
Yield strength (MPa)	589	942	589	570
Ductility (%)	1.9	7.8	1.7	2.4
Fracture toughness (MPa·m^1/2^)	23	52	23	20
Thermal conductivity (W/(m·K))	24	8.6	24	19
Maximum operating temperature (°C)	900	615	900	865

**Table 2:** Machining conditions for the 8.5 mm hole.

Material	*v_c_* [m/min]	*n* [rev/min]	*f* [mm/rev]	*f* [mm/rev]
Ti-6Al-4V	50	1874	0.05	0.1
TNB	15	1874	0.05	0.1
MoCuSi Extruded	15	562	0.05	0.1
MoCuSi Ingot	15	562	0.05	0.1

**Table 3:** Properties of Rhenus^®^ FU 70 W coolant.

Concentrated		Emulsion	
Viscosity 20 °C (mm^2^/s)	Content of mineral oil %	pH Value 5% concentration	Protection against corrosion (DIN 51360/1)
Approx. 150	Approx. 33	Approx. 9,0	Note 0 al 2%

**Table 4:** Experimental results for *F_z_* [N] and *T_c_* [Nm] in Ti-6Al-4V alloy.

*D*_0_ [mm]	*F_z_* [N]		*T_c_* [Nm]	
	*f* [mm/rev]		*f* [mm/rev]	
	0.05	0.1	0.05	0.1
3	591	723	3.1	3.9
4	460	565	2.4	3.7
5	319	440	2.1	3.4
6	242	332	1.9	3.2
7	169	225	1.8	3.1

**Table 5:** Experimental results for *F_z_* [N] and *T_c_* [Nm] in TNB alloy.

*D*_0_ [mm]	*F_z_* [N]		*T_c_* [Nm]	
	*f* [mm/rev]		*f* [mm/rev]	
	0.05	0.1	0.05	0.1
3	802	954	5.8	7.0
4	631	765	5.3	6.7
5	476	575	4.3	6.1
6	343	421	4.0	6.0
7	240	259	3.5	5.7

**Table 6:** Experimental results for *F_z_* [N] and *T_c_* [Nm] in ingot MoCuSi alloy.

*D*_0_ [mm]	*F_z_* [N]		*T_c_* [Nm]	
	*f* [mm/rev]		*f* [mm/rev]	
	0.05	0.1	0.05	0.1
3	852	906	6.0	8.7
4	702	775	5.5	8.3
5	525	600	4.8	7.5
6	419	466	4.6	7.3
7	275	290	4.0	7.0

**Table 7:** Experimental results for *F_z_* [N] and *T_c_* [Nm] in extruded MoCuSi alloy.

*D*_0_ [mm]	*F_z_* [N]		*T_c_* [Nm]	
	*f* [mm/rev]		*f* [mm/rev]	
	0.05	0.1	0.05	0.1
3	925	1050	6.5	9.3
4	751	860	6.1	8.6
5	550	621	5.5	8.0
6	427	475	4.7	7.7
7	280	292	4.0	7.5

**Table 8:** Obtained coefficients for the polynomials in Equations (3) and (4).

Materials	*K_z_(h,r)*						*K_T_(h,r)*					
	a_0_	a_1_	a_2_	a_3_	a_4_	a_5_	b_0_	b_1_	b_2_	b_3_	b_4_	b_5_
Ti-6Al-4V	13,159	−93,861	−3,225	−10,986	977.11	−7,056.0	39.646	−68.280	−21.597	−41.882	5.253	−5.171
TNB	19,860	−217,630	−3,830	17,235	855.42	−16,347.1	77.853	−283.109	−36.204	−61.168	8.334	−21.331
MoCuSi (ingot)	23,445	−270,543	−4,028	20,962	688.07	−20,320	90.152	−274.538	−45.120	−17.808	9.663	−20.703
MoCuSi (extruded)	24,016	−282,289	−4,270	24,114	860.73	−21,202	84.201	−120.895	−45.120	−17.808	9.663	−20.703

**Table 9:** Cutting conditions for the validation tests.

Materials	*V_c_* [m/min]	*D*_0_ [m/min]	*f* [mm/rev]	*# Test*
Ti-6Al-4V	50	4	0.06	1
		6	0.08	2
TNB	15	4	0.06	3
		6	0.08	4
MoCuSi (ingot)	15	4	0.06	5
		6	0.08	6
MoCuSi (extruded)	15	4	0.06	7
		6	0.08	8

## References

[1] Homepage of Boeing. http://www.boeing.com.

[2] Martin R., Evans D. (2000). Reducing Costs in Aircraft: The Metals Affordability Initiative Consortium. JOM.

[3] Voice W.E., Henderson M., Shelton E.F.J., Wu X. (2005). Gamma titanium aluminide-TNB. Intermetallics.

[4] Boyer R.R. (1996). An overview on the use of titanium in the aerospace industry. Mater. Sci. Eng. A.

[5] Jha A.K., Singh S.K., Kiranmayee M.S., Sreekumar K., Sinha P.P. (2010). Failure analysis of titanium alloy (Ti6Al4V) fastener used in aerospace application. Eng. Fail. Anal..

[6] Murr L.E., Quinones S.A., Gaytan S.M., López M.I., Rodela A., Martinez E.Y., Hernandez D.H., Martinez E., Medina F., Wicker R.B. (2009). Microstructure and mechanical behavior of Ti-6Al-4 V produced by rapid-layer manufacturing for biomedical applications. J. Mech. Behav. Biomed. Mater..

[7] Yamaguchi M., Inui H., Ito K. (2000). High temperature structural intermetallics. Acta Mater..

[8] Jozwik J. (2018). Evaluation of Tribological Properties and Condition of Ti6Al4V Titanium Alloy Surface. Tehnički vjesn..

[9] Kim Y.W., Dimiduk D.M. Gamma TiAl alloys: Emerging new structural metallic materials. Proceedings of the ICETS 2000-ISAM.

[10] Semiatin S.L., Seetharaman V., Weiss I. (1998). Hot workability of titanium and titanium aluminide alloys. Mater. Sci. Eng..

[11] López de Lacalle L.N., Perez J., Llorente J.I., Sanchez J.A. (2000). Advanced cutting conditions for the milling of aeronautical alloys. J. Mater. Process. Technol..

[12] Kuczmaszewski J., Kazimierz Z., Matuszak J., Pałka T., Mądry J. (2017). Studies on the effect of mill microstructure upon tool life during slot milling of Ti6Al4V alloy parts. Eksploat. I Niezawodn. Maint. Reliab..

[13] Józwik J., Ostrowski D., Milczarczyk R., Krolczyk G.M. (2018). Analysis of relation between the 3D printer laser beam power and the surface morphology properties in Ti-6Al-4V titanium alloy parts. J. Braz. Soc. Mech. Sci. Eng..

[14] Aspinwall D.K., Dewes R.C., Mantle A.L. (2005). The Machining of γ-TiAl Intermetallic Alloys. CIRP Ann..

[15] Arrazola P.J., Garay A., Iriarte L.M., Armendia M., Marya S., Maitre F.L. (2009). Machinability of titanium alloys (Ti6Al4V and Ti555.3). J. Mater. Process. Technol..

[16] Beranoagirre A., López de Lacalle L.N. (2013). Grinding of Gamma TiAl Intermetallic Alloys. Proc. Eng..

[17] Lindemann J., Glavatskikh M., Leyens C. Surface effects on the mechanical properties of gamma titanium aluminides. Proceedings of the International Conference on Processing & Manufacturing of Advanced Materials.

[18] Hood T., Aspinwall D.K., Sage C., Voice W. (2013). High speed ball nose and milling of γ-TiAl alloys. Intermetallics.

[19] Thepsonthi T., Ozel T. (2013). Experimental and Finite Element Simulation based Investigations on Micro-Milling Ti–6Al–4V Titanium Alloy: Effects of cBN Coating on Tool Wear. J. Mater. Process. Technol..

[20] Cantero J.L., Tardio M.M., Canteli J.A., Marcos M., Miguelez M.H. (2005). Dry drilling of alloy Ti6Al4V. Int. J. Mach. Tools Manuf..

[21] Hood R., Aspinwall D.K., Soo S.L., Mantle A.L., Novovic D. (2014). Workpiece surface integrity when slot milling γ-TiAl intermetallic alloy. CIRP Ann..

[22] Liu J., Zhu D., Zhao L., Xu Z. (2015). Experimental Investigation on Electrochemical Machining of γ-TiAl Intermetallic. Proc. CIRP.

[23] Kosaraju S., Anne V.G. (2013). Optimal machining conditions for turning Ti-6Al-4 V using response surface methodology. Adv. Manuf. Technol..

[24] Mantle A.L., Aspinwall D.K. Temperature Measurement and Tool Wear When turning gamma titanium aluminide intermetallic. Proceedings of the 13th Conference of the Irish Manufacturing Committee (IMC-13).

[25] Aspinwall D.K., Mantle A.L., Chan W.K., Hood R., Soo S.L. (2013). Cutting Temperatures when Ball Nose End Milling γ-TiAl Intermetallic Alloys. CIRP Ann..

[26] Mantle A.L., Aspinwall D.K. (2001). Surface integrity of a high speed milled gamma titanium aluminide. J. Mater. Process. Technol..

[27] Olvera D., Urbicain G., Beranoagirre A., López de Lacalle L.N., Katalinic B. (2010). Hole Making in Gamma TiAl. DAAAM International Scientific Book 2010.

[28] Priarone P.C., Rizzuti S., Settineri L., Vergnano G. (2012). Effects of Cutting Angle, Edge Preparation and Nano-Structured Coating on Milling Performance of a Gamma Titanium Aluminide. J. Mater. Process. Technol..

[29] Hong S.Y., Ding Y. (2001). Cooling approaches and cutting temperatures in cryogenic machining of Ti-6Al-4 V. Int. J. Mach. Tools Manuf..

[30] Marasi A. (2013). Modeling the effects of cutting parameters on the main cutting force of Ti6Al4V alloy by using hybrid approach. Int. J. Adv. Eng. Appl..

[31] Niesłony P., Grzesik W., Laskowski P., Sienawski J. (2014). Numerical and Experimental Analysis of Residual Stresses Generated in the Machining of Ti6Al4V Titanium Alloy. Procedia CIRP.

[32] Kadirgama K., Abou-El-Hossein K.A., Mohammad B., Habeeb A., Noor M.M. (2008). Cutting force prediction model by FEA and RSM when machining Hastelloy C-22HS with 90° holder. J. Sci. Ind. Res..

[33] Bagci E., Ozcelik B. (2006). Finite element and experimental investigation of temperature changes on a twist drill in sequential dry drilling. Int. J. Adv. Manuf. Technol..

[34] Chatterjee S., Mahapatra S.S., Abhishek K. (2016). Simulation and optimization of machining parameters in drilling of titanium alloys. Simul. Model. Pract. Theory.

[35] Franco A., Abul C., Rashed A., Romoli L. (2016). Analysis of energy consumption in micro-drilling processes. J. Clean. Prod..

[36] Hameed S., Rojas H.A.G., Egea A.J.S., Alberro A.N. (2016). Electroplastic cutting influence on power consumption during drilling process. Int. J. Adv. Manuf. Technol..

[37] Azeem A., Feng H.Y., Wang L. (2004). Simplified and efficient calibration of a mechanistic cutting force model for ball-end milling. Int. J. Mach. Tools Manuf..

[38] Altintas Y. (2000). Manufacturing Automation: Metal Cutting Mechanics, Machine Tool Vibrations, and CNC Design.

[39] Budak E., Altintas Y., Armarego E.J.A. (1996). Prediction of milling force coefficients from orthogonal cutting data. ASME J. Manuf. Sci. Eng..

[40] Roukema J.C., Altintas Y. (2006). Time domain simulation of torsional–axial vibrations in drilling. Int. J. Mach. Tools Manuf..

[41] Beranoagirre A., Olvera D., López de Lacalle L.N., Urbicain G. (2011). Drilling of intermetallic alloys gamma TiAl. AIP Conf. Proc..

[42] Beranoagirre A., Urbicain G., Calleja A., López de Lacalle L.N. (2018). Hole Making by Electrical Discharge Machining (EDM) of γ-TiAl Intermetallic Alloys. Metals.

